# Associations of cumulative occupational benzene exposure with genotoxic and immunosenescence-related biomarkers: a cross-sectional study

**DOI:** 10.3389/fpubh.2026.1972556

**Published:** 2026-10-14

**Authors:** Wenqian Xu, Xiaoying Ruan, Lu He, Yaying Zhang, Yun Sun

**Affiliations:** 1 Department of Party and Mass Affairs, Shanghai Yangpu District Center for Disease Control and Prevention (Shanghai Yangpu District Health Supervision Institute), Shanghai, China; 2 Department of Occupational Health, Hangzhou Hospital for Prevention and Treatment of Occupational Disease, Hangzhou, China; 3 Shanghai Yangpu District Center for Health Promotion, Shanghai, China; 4 Department of Environmental and Occupational Health, Shanghai Yangpu District Center for Disease Control and Prevention (Shanghai Yangpu District Health Supervision Institute), Shanghai, China; 5 Department of Physical Examination, Shanghai Institute of Occupational Disease for Chemical Industry, Shanghai, China

**Keywords:** benzene, comet assay, genotoxicity, immunosenescence, micronucleus, occupational exposure

## Abstract

**Background:**

Chronic benzene (BZ) exposure is a well-established cause of hematological malignancies, but its potential association with immunosenescence-related alterations at occupational exposure levels below current regulatory limits remains unclear. This cross-sectional study investigated the associations of cumulative BZ exposure with genotoxicity, immunosenescence-related, and hematological biomarkers.

**Methods:**

The study included 228 participants: 115 workers directly involved in BZ production, 75 administrative workers from the same factories without direct involvement in BZ production, and 38 external controls without occupational exposure to industrial solvents. BZ exposure was assessed using personal air monitoring, and cumulative exposure dose (CED) was calculated from airborne BZ concentrations and exposure duration. Genomic damage was evaluated using the cytokinesis-block micronucleus (CBMN) and comet assays. Immunosenescence-related and cellular-stress markers included the CD4/CD8 ratio, CD28^−^ frequency, p53 expression, *γ*-H2AX expression, and mitochondrial DNA copy number. Hematological parameters were assessed using peripheral blood cell counts. Spearman correlation analysis and outcome-appropriate generalized linear models were used to evaluate the associations between CED and the investigated biomarkers.

**Results:**

After full multivariable adjustment, higher CED was associated with greater comet tail intensity (MR = 1.0827, *p* < 0.001) and tail moment (MR = 1.0922, *p* < 0.001) and with lower CD4/CD8 ratio (MR = 0.9743, *p* < 0.001) and p53 expression (MR = 0.9739, *p* < 0.001). CED was associated with higher micronucleus frequency (MNF) in the age- and sex-adjusted model (RR = 1.0137, *p* = 0.033). After further adjustment for BMI, hypertension, smoking status, and alcohol use, the effect estimate remained positive, but the association did not reach conventional statistical significance (RR = 1.0123, *p* = 0.053). No statistically significant associations were observed between CED and comet tail length, CD28^−^ frequency, *γ*-H2AX expression, mitochondrial DNA copy number, WBC, RBC, platelet count, or neutrophil count in the fully adjusted models.

**Conclusion:**

Cumulative occupational BZ exposure below the applicable regulatory limits was associated with increased comet-assessed DNA damage and selected immune-related alterations, particularly lower CD4/CD8 ratio and p53 expression. The association between CED and MNF remained positive but was not statistically significant after full adjustment. These findings suggest a selective pattern of BZ-associated genotoxic and immune-related alterations rather than generalized hematological toxicity or a uniform immunosenescence phenotype. Prospective studies are needed to determine their long-term clinical and occupational health significance.

## 1 Introduction

Benzene (BZ), a volatile organic compound and a foundational petrochemical, remains a significant occupational and environmental health concern (1). BZ is classified by the International Agency for Research on Cancer (IARC) as carcinogenic to humans (Group 1) (2) and is causally associated with acute myeloid leukemia (AML); associations with several other hematological malignancies have also been reported (3, 4). Occupational exposure to BZ remains a concern in industries including petroleum refining, chemical manufacturing, shoe production, and gasoline or fuel distribution (5). The hematopoietic system, particularly the bone marrow, is a major target of BZ toxicity (1, 6). Bone marrow is the principal site of adult hematopoiesis and immune-cell development (7). Severe BZ exposure can cause profound bone-marrow suppression, including pancytopenia and aplastic anemia (1, 8), whereas chronic occupational exposure is causally associated with AML (3, 4, 6). Benzene is metabolized to reactive metabolites, including hydroquinone, which contribute to genotoxicity, oxidative stress, altered hematopoietic stem and progenitor cell function, and cell death (1, 6).

Traditionally, occupational safety standards for BZ have been established based on the prevention of these overt hematological disorders and cancer (9). Regulatory limits, such as the OSHA Permissible Exposure Limit (PEL) of 1 ppm as an 8-h time-weighted average, are primarily anchored to this carcinogenic risk (9, 10). However, a growing body of epidemiological evidence suggests that chronic, low-level exposure to BZ, at concentrations below these regulatory limits, is associated with subtler, yet significant, hematotoxic effects (11–13). These subclinical effects include reduced peripheral blood cell counts—particularly neutrophils, lymphocytes and platelets—in workers with low-level exposure, consistent with bone-marrow toxicity even below current occupational limits (12–14).

Early hematological changes after BZ exposure include both decreased and, in some contexts, altered leukocyte subset counts, together with differential expression of genes related to DNA damage response and repair. Whether these changes reflect a sustained immune-system imbalance remains under investigation (11, 15, 16). Consequently, it is difficult to obtain definitive biomarkers of early or low-dose BZ exposure in humans. Immune cell alterations have been reported in BZ-exposed populations; however, it remains unclear whether these cells are primarily damaged by BZ metabolites or undergo senescence-like changes under chronic stress (16, 17). Parallel to these developments in BZ toxicology, the field of immunology has increasingly recognized immunosenescence as a critical determinant of healthspan (18, 19). Immunosenescence is not merely a passive consequence of chronological aging but a complex remodeling of the immune system, characterized by reduced regenerative capacity and a shift toward a chronic pro-inflammatory state (18–20). This senescent immune phenotype is clinically significant, as it is strongly associated with increased vulnerability to infections, poor response to vaccines, higher rates of autoimmune conditions, and overall increased morbidity and mortality in older adults (19, 21, 22).

An under-explored hypothesis is that certain environmental toxicants may accelerate immune aging, causing immune age to diverge from chronological age (23–27). Given the well-established hematopoietic and bone-marrow toxicity of BZ, including effects on hematopoietic progenitor-cell compartments, BZ is a biologically plausible candidate environmental modifier of immune aging (12, 17). Maintenance of a diverse and functional T-cell pool depends on the generation of bone-marrow-derived lymphoid progenitors, thymic production of naïve T cells, and homeostatic maintenance of peripheral T-cell populations (19) By damaging HSPCs and the bone marrow microenvironment (28, 29), chronic BZ exposure could theoretically disrupt this homeostatic process in several ways: by directly inducing senescence or apoptosis in lymphoid progenitors, by forcing premature proliferative exhaustion in surviving clones to maintain peripheral counts, or by inducing a chronic inflammatory state that drives terminal differentiation of T-cells (6, 12, 22, 30).

Preliminary evidence suggests that BZ-exposed workers may exhibit altered hematological profiles and markers of B-cell activation, together with changes in oxidative-stress and inflammation-related signaling pathways (16, 17, 31). To our knowledge, few studies have comprehensively integrated quantitative BZ exposure assessment with hematological indices, genomic damage, and multiple immunosenescence-related biomarkers. In particular, the potential associations of chronic occupational BZ exposure with T-cell homeostasis and cellular stress responses remains insufficiently characterized. We therefore hypothesized that cumulative BZ exposure would be associated with alterations in genomic stability, hematological parameters, and immunosenescence-related markers. If confirmed, such findings would suggest that the long-term public-health implications of BZ exposure may extend beyond malignancy and overt hematotoxicity to include subclinical immune-related alterations.

## 2 Methods and materials

### 2.1 Study population and design

A cross-sectional study was conducted among 228 adult participants, who were classified into three groups according to their occupational roles and potential for BZ exposure. The exposed group comprised 115 workers who had been directly involved in pure BZ production for at least 5 years. The internal control group comprised 75 administrative workers from the same factories who were not directly involved in BZ production but performed routine inspections of the production areas. The external control group comprised 38 administrative personnel with no history of occupational exposure to BZ or other industrial solvents. Exclusion criteria for all participants included: history of chemotherapy or radiotherapy, hepatitis B/C, active tuberculosis, or other known immunodeficiencies, current use of immunosuppressive medications, recent blood transfusion (<6 months), and diagnosis of hematological malignancies or solid tumors. All participants provided written informed consent. The study protocol was approved by the Institutional Review Board of Shanghai Yangpu District Center for Disease Control and Prevention (Shanghai Yangpu District Health Supervision Institute).

### 2.2 Exposure assessment

Personal exposure to BZ was assessed using passive diffusion air samplers worn for a full 8-h shift by both exposed workers and internal controls. Time-weighted average (TWA) concentrations were calculated in parts per million (ppm). For each participant, a 2 mL fresh blood sample preserved with heparin was collected, and hematological parameters were subsequently measured. The BZ-exposed workers were engaged in the production of pure BZ, and their exposure levels were obtained from available environmental monitoring data covering the past 10 years. Cumulative exposure doses (CEDs) for these workers were further calculated using the formula: CED (mg/m^3^·year) = Σ [C (mg/m^3^) × T (year)], where C represents the airborne BZ concentration at the worksite and T denotes the exposure duration for workers at different posts.

### 2.3 Cytokinesis-blocked micronucleus (CBMN) assay

0.5 mL Peripheral blood samples were collected into heparin-preserved tubes from each participant. The whole blood was cultured in RPMI 1640 medium supplemented with phytohemagglutinin and fetal bovine serum for 48 h. Cytochalasin B (final concentration 6 μg/mL) was then added to block cytokinesis, and the incubation continued for an additional 24 h. Cells were harvested, treated with hypotonic KCl solution at 37 °C for 30 min, fixed three times in a pre-cooled methanol/acetic acid mixture (3:1, v/v), dropped onto glass slides, and air-dried overnight. Slides were stained with Giemsa solution (diluted 1:9 in PBS) for 20 min. For each participant, 1,000 binucleated cells were scored under a light microscope to determine the micronucleus frequency (MNF), expressed as the number of micronuclei per 1,000 binucleated cells.

### 2.4 Assessment of genotoxicity: comet assay

For the comet assay (single-cell gel electrophoresis), 20 μL of fresh heparinized blood was mixed with 180 μL of low-melting-point agarose at 37 °C and immediately dropped onto glass slides pre-coated with normal-melting-point agarose. Slides were placed at 4 °C for 30 min and then immersed in lysis solution at 4 °C overnight. Subsequently, slides were subjected to alkaline electrophoresis (pH > 13) at a constant current of 300 mA for 20 min at low temperature. After neutralization and staining with ethidium bromide, comet images were captured using an inverted fluorescence microscope. Each slide was analyzed twice using Comet assay software (Comet IV). In each analytical run, one hundred randomly selected cells were evaluated, and the software directly calculated the mean comet tail intensity, tail length, and tail moment across the 100 cells. Comet tail intensity was expressed as the percentage of DNA in the comet tail, tail length was defined as the distance from the center of the comet head to the end of the tail (μm), and tail moment was calculated as the product of tail length and tail intensity. For each participant, the mean of the values obtained from the two analytical runs was calculated for each comet parameter and used as the participant-level value in the statistical analyses.

### 2.5 Assessment of immunosenescence-related biomarkers

Peripheral blood (2 mL) was drawn from each participant into EDTA tubes. Peripheral blood mononuclear cells (PBMCs) were isolated within 4 h using Ficoll-Paque PLUS density gradient centrifugation. T-cell immunophenotyping was performed using multicolor flow cytometry (BD FACSCanto II) with fluorochrome-conjugated antibodies against CD4, CD8, and CD28. The frequencies of CD28^−^ cells within the CD4+ and CD8+ T-cell populations were determined, and the CD4/CD8 ratio was calculated.

### 2.6 Evaluating p53 level by quantitative real-time PCR

We employed quantitative real-time PCR (qRT-PCR) to determine the expression level of p53 in total RNA extracted from whole blood. Briefly, whole blood samples were thoroughly lysed in three volumes of TRIzol^®^ reagent, and RNA was subsequently isolated. The concentration of RNA from each individual sample was measured, and the reverse-transcription and qRT-PCR reaction mixtures were prepared according to the manufacturer’s protocol for the two-step procedure. Amplification and fluorescence detection were performed on a BD real-time PCR system. Relative p53 expression was calculated using the 2^ΔΔCt^ method. Each sample was assayed in triplicate, and the mean value was used as the final expression level for subsequent analyses.

### 2.7 H2AX detection by flow cytometry

PBMCs were isolated from 1 mL of whole blood via Ficoll-Paque density gradient centrifugation. After washing, the cells were permeabilized and stained with FITC-labeled anti-*γ*-H2AX antibody for 1 h at room temperature. Following washing, *γ*-H2AX expression was determined by flow cytometry, with a minimum of 10,000 events acquired per sample, and data were analyzed using FlowJo software.

### 2.8 Statistical analysis

Data were analyzed using IBM SPSS Statistics version 26.0. The normality of continuous variables was assessed using the Shapiro–Wilk test. Continuous variables with approximately normal distributions were summarized as mean ± standard deviation (SD), whereas non-normally distributed variables were summarized as median (Q1–Q3). Group differences in continuous variables were assessed using one-way ANOVA or the Kruskal-Wallis test, as appropriate, followed by *post-hoc* tests with Bonferroni correction for multiple comparisons. Categorical variables were summarized as *n* (%) and compared using the Chi-square test. Unadjusted associations between cumulative benzene exposure dose (CED) and the investigated biomarkers were evaluated using Spearman rank correlation analysis. Generalized linear models were used to evaluate the multivariable associations between CED and each outcome. Model 1 was adjusted for age and sex, and Model 2 was additionally adjusted for BMI, hypertension, smoking status, and alcohol use. Both models were fitted using participants with complete data for all included covariates to ensure a consistent analytical sample. Model distributions and link functions were selected according to the measurement scale and distributional characteristics of the outcomes. Micronucleus (MN) counts were analyzed using a negative binomial model with a log link because they exhibited overdispersion relative to a Poisson distribution; the logarithm of the number of binucleated cells scored was included as an offset. Positively skewed continuous outcomes were analyzed using Gamma models with a log link, whereas approximately normally distributed continuous outcomes were analyzed using Gaussian models with an identity link. The negative binomial ancillary parameter was estimated using maximum likelihood, and the scale parameters for the Gamma and Gaussian models were estimated using the Pearson chi-square method. Results from negative binomial models are presented as rate ratios (RRs), results from gamma models as mean ratios (MRs), and results from Gaussian models as unstandardized regression coefficients (B), with corresponding 95% confidence intervals (CIs). RRs and MRs represent the relative change in the corresponding outcome associated with a 1-mg/m^3^·year increase in CED, whereas B represents the absolute change associated with the same increase in CED. All tests were two-sided, and *p* < 0.05 was considered statistically significant.

## 3 Results

### 3.1 Participant characteristics and biomarker levels

Table 1 presents characteristics and biomarker levels of study participants stratified by exposure group: external control (*n* = 38), internal control (*n* = 75), and exposed group (*n* = 115). Group allocation was determined by a combination of job tenure and actual monitored benzene concentrations at the workplace.

**Table 1:** Characteristics and biomarker levels of study participants by study group.

Characteristic	External control	Internal control	Exposed group	*p-*value
	(*n* = 38)	(*n* = 75)	(*n* = 115)	
Age, year	38.50 (34.00–46.00)	41.00 (33.00–47.00)	46.00 (41.00–54.00)	<0.001
Working age, year	17.00 (13.00–26.25)	21.00 (10.00–27.00)	23.00 (21.00–34.00)	<0.001
CED, mg/m^3^·year	0.00 (0.00–0.00)	2.83 (1.48–3.57)	9.67 (5.16–13.13)	<0.001
MNF, MN per 1,000 binucleated cells	4.00 (3.00–6.00)	8.00 (5.00–12.00)	9.00 (7.00–11.00)	<0.001
Comet tail intensity, % tail DNA	0.82 (0.25–1.46)	0.47 (0.12–1.33)	1.65 (0.47–3.90)	<0.001
Comet tail length, μm	19.32 ± 4.75	20.28 ± 4.19	20.63 ± 4.12	0.256
Comet tail moment	0.07 (0.02–0.15)	0.04 (0.01–0.13)	0.19 (0.04–0.41)	<0.001
CD28^−^ frequency	0.24 ± 0.04	0.25 ± 0.05	0.24 ± 0.05	0.218
CD4/CD8 ratio	3.04 (2.31–3.95)	1.29 (1.00–1.56)	1.01 (0.83–1.31)	<0.001
p53 expression	1.75 (1.50–1.99)	0.58 (0.51–0.76)	0.52 (0.44–0.63)	<0.001
*γ*-H2AX expression	2.42 (1.92–3.19)	2.35 (1.89–3.24)	2.28 (1.82–2.86)	0.399
mtDNA copy number	5.62 ± 0.61	5.86 ± 0.85	5.68 ± 0.86	0.216
WBC, *10^9^/L	6.11 ± 1.47	6.50 ± 1.41	6.08 ± 1.28	0.093
RBC, *10^12^/L	4.92 ± 0.43	5.02 ± 0.34	4.91 ± 0.31	0.089
PLT, *10^9^/L	253.26 ± 42.11	202.68 ± 44.29	205.13 ± 45.74	<0.001
NEUT, *10^9^/L	3.49 (3.15–4.89)	5.40 (4.80–6.00)	5.20 (4.70–5.90)	<0.001
Sex				<0.001
Male	21 (55.26)	69 (92.00)	115 (100.00)	
Female	17 (44.74)	6 (8.00)	0 (0.00)	
BMI, kg/m^2^	23.78 ± 3.26	23.83 ± 3.00	24.38 ± 3.46	0.425
Smoking status	5 (13.16)	41 (54.67)	70 (60.87)	<0.001
Alcohol use	38 (100.00)	42 (56.00)	70 (60.87)	<0.001
Hypertension	4 (10.53)	16 (21.33)	24 (20.87)	0.323

Data are presented as mean ± SD, median (Q1–Q3), or *n* (%), as appropriate.

CED, cumulative exposure dose; MNF, micronucleus frequency; mtDNA, mitochondrial DNA; WBC, white blood cell count; RBC, red blood cell count; PLT, platelet count; NEUT, neutrophil count; BMI, body mass index.

Regarding genotoxicity and hematological markers, BZ exposure was notably associated with an increase in MNF, escalating from 4.00 (3.00–6.00) in external control to 9.00 (7.00–11.00) in exposed group. Similar upward trends were observed in comet assay parameters (comet tail intensity and comet tail moment) among exposed group. For immune and apoptotic markers, the CD4/CD8 ratio dramatically declined in exposed workers [1.29 (1.00–1.56) and 1.01 (0.83–1.31)] in internal control and exposed group, respectively compared to the external control [3.04 (2.31–3.95)]. Correspondingly, p53 levels were profoundly depressed in exposed individuals [0.58 (0.51–0.76) and 0.52 (0.44–0.63)] relative to the control baseline [1.75 (1.50–1.99)]. Furthermore, a marked reduction in platelet (PLT) counts was observed in both exposed cohorts (~202–205) compared to external control (253.26 ± 42.11). Sex and lifestyle factors such as smoking and alcohol use varied significantly across groups, whereas body mass index (BMI) and the prevalence of hypertension remained relatively stable across all strata.

### 3.2 Correlations of cumulative benzene exposure dose with the investigated biomarkers

Spearman rank correlation analysis was performed to examine the unadjusted relationships between CED and the investigated biomarkers. CED was positively correlated with MNF (*r*ₛ = 0.3632, *p* < 0.001), comet tail intensity (*r*ₛ = 0.3084, *p* < 0.001), and tail moment (*r*ₛ = 0.3365, *p* < 0.001), and negatively correlated with the CD4/CD8 ratio (*r*ₛ = −0.5267, *p* < 0.001) and p53 expression (*r*ₛ = −0.5914, *p* < 0.001). CED was also weakly correlated with comet tail length, PLT, and neutrophil count (NEUT). No statistically significant correlations were observed between CED and CD28^−^ frequency, *γ*-H2AX expression, mitochondrial DNA (mtDNA) copy number, WBC, or RBC (Table 2).

**Table 2:** Spearman correlations between cumulative benzene exposure dose and the investigated biomarkers.

Biomarkers	Spearman’s *r*_s_	*p*-value
MNF	0.3632	<0.001
Comet tail intensity	0.3084	<0.001
Comet tail length	0.1423	0.032
Comet tail moment	0.3365	<0.001
CD4/CD8 ratio	−0.5267	<0.001
CD28^−^ frequency	−0.0922	0.165
p53 expression	−0.5914	<0.001
*γ*-H2AX expression	−0.1227	0.064
mtDNA copy number	−0.0099	0.882
WBC	−0.0208	0.755
RBC	−0.1021	0.124
PLT	−0.2290	0.001
NEUT	0.2648	0.0001

MNF, micronucleus frequency; mtDNA, mitochondrial DNA; WBC, white blood cell count; RBC, red blood cell count; PLT, platelet count; NEUT, neutrophil count.

### 3.3 Multivariable associations between cumulative benzene exposure dose and the investigated biomarkers

In the negative binomial model of MN counts, higher CED was associated with a higher MNF in Model 1 (RR = 1.0137, 95% CI 1.0011–1.0264, *p* = 0.033). After full adjustment, the effect estimate remained positive, but the association did not reach conventional statistical significance (RR = 1.0123, 95% CI 0.9998–1.0248, *p* = 0.053).

CED was consistently associated with greater comet tail intensity and tail moment. In the fully adjusted models, each 1-mg/m^3^·year increase in CED was associated with an 8.27% higher mean tail intensity (MR = 1.0827, 95% CI 1.0472–1.1194, *p* < 0.001) and a 9.22% higher mean tail moment (MR = 1.0922, 95% CI 1.0518–1.1343, *p* < 0.001). No statistically significant association was observed between CED and tail length after full adjustment (*B* = 0.0575, 95% CI − 0.0552–0.1703, *p* = 0.317).

For immune- and stress-related biomarkers, higher CED was associated with a lower CD4/CD8 ratio (MR = 0.9743, 95% CI 0.9645–0.9843, *p* < 0.001) and lower p53 expression (MR = 0.9739, 95% CI 0.9661–0.9818, *p* < 0.001) after full adjustment. CED was not significantly associated with CD28^−^ frequency, *γ*-H2AX expression, or mtDNA copy number in either model. Among the hematological parameters, NEUT was positively associated with CED in Model 1, but this association was attenuated after full adjustment. No statistically significant associations with CED were observed for WBC, RBC, PLT, or NEUT in the fully adjusted models (Table 3).

**Table 3:** Associations between cumulative benzene exposure dose and the investigated biomarkers estimated using generalized linear models.

Outcome	Model and effect measure	Model 1 estimate (95% CI)	*p-*value	Model 2 estimate (95% CI)	*p*-value
MNF	Negative binomial, RR	1.0137 (1.0011–1.0264)	0.033	1.0123 (0.9998–1.0248)	0.053
Comet tail intensity	Gamma, MR	1.0823 (1.0472–1.1186)	<0.001	1.0827 (1.0472–1.1194)	<0.001
Comet tail length	Gaussian, B	0.0744 (−0.0377–0.1866)	0.193	0.0575 (−0.0552–0.1703)	0.317
Comet tail moment	Gamma, MR	1.0940 (1.0523–1.1373)	<0.001	1.0922 (1.0518–1.1343)	<0.001
CD4/CD8 ratio	Gamma, MR	0.9709 (0.9602–0.9817)	<0.001	0.9743 (0.9645–0.9843)	<0.001
p53 expression	Gamma, MR	0.9671 (0.9575–0.9759)	<0.001	0.9739 (0.9661–0.9818)	<0.001
CD28^−^ frequency	Gaussian, B	−0.0008 (−0.0021–0.0005)	0.224	−0.0004 (−0.0014–0.0005)	0.364
*γ*-H2AX expression	Gamma, MR	1.0037 (0.9952–1.0124)	0.392	1.0016 (0.9975–1.0057)	0.444
mtDNA copy number	Gaussian, B	−0.0086 (−0.0301–0.0129)	0.433	−0.0087 (−0.0304–0.0130)	0.431
WBC	Gaussian, B	−0.0240 (−0.0595–0.0115)	0.186	−0.0344 (−0.0691–0.0003)	0.052
RBC	Gaussian, B	−0.0040 (−0.0129–0.0049)	0.373	−0.0046 (−0.0137–0.0044)	0.314
PLT	Gaussian, B	−1.0743 (−2.3023–0.1536)	0.086	−0.9276 (−2.1496–0.2944)	0.137
NEUT	Gamma, MR	1.0075 (1.0005–1.0146)	0.036	1.0058 (0.9990–1.0127)	0.095

RR, rate ratio; MR, mean ratio; B, unstandardized regression coefficient; MNF, micronucleus frequency; mtDNA, mitochondrial DNA; WBC, white blood cell count; RBC, red blood cell count; PLT, platelet count; NEUT, neutrophil count.

Model 1 was adjusted for age and sex. Model 2 was further adjusted for BMI, hypertension, smoking status, and alcohol use.

Effect estimates are presented as RRs for negative binomial models with a log link, MRs for gamma models with a log link, and unstandardized regression coefficients for Gaussian models with an identity link. All estimates represent the change in the corresponding outcome associated with a 1-mg/m^3^·year increase in CED. For MNF, the logarithm of the number of binucleated cells scored was included as an offset.

## 4 Discussion

This cross-sectional study examined the associations of cumulative occupational BZ exposure with markers of genotoxicity, including DNA damage and chromosomal instability, as well as with hematological and immunosenescence-related biomarkers. Although occupational BZ exposure levels were below the applicable regulatory limit, the fully adjusted analyses indicated a selective pattern of comet-assessed DNA damage and immune-related alterations, without consistent associations with routine peripheral blood counts. Overall, these findings suggest selective genotoxic and immune-related alterations rather than generalized hematological toxicity or a uniform immunosenescence phenotype.

The most consistent evidence of genotoxicity in our study was provided by the comet assay. Higher CED was associated with greater comet tail intensity and tail moment in both the age- and sex-adjusted and fully adjusted models, whereas no statistically significant association was observed for comet tail length. MNF, another marker of chromosomal instability (32), was positively correlated with CED in the Spearman analysis and was associated with CED in the age- and sex-adjusted model. After further adjustment, the effect estimate remained positive, but the association did not reach conventional statistical significance (RR = 1.0123, *p* = 0.053). BZ metabolites, including hydroquinone (HQ) and 1,4-benzoquinone (BQ), induce reactive oxygen species (ROS) generation and form covalent DNA adducts, resulting in oxidative DNA lesions and DNA strand breaks, particularly in hematopoietic stem and progenitor cells (33, 34). Accordingly, the persistent associations with comet tail intensity and tail moment provide the primary evidence of exposure-related DNA damage in this study, whereas the MNF result should be considered supportive rather than definitive evidence of chromosomal damage.

Concurrent with the genotoxicity findings, higher CED was associated with a lower CD4/CD8 ratio and lower p53 expression in both the age- and sex-adjusted and fully adjusted models. A reduced CD4/CD8 ratio is a recognized indicator of altered T-cell homeostasis, and ratio inversion (CD4/CD8 < 1) has been described as a component of the immune risk profile associated with chronic viral infection, particularly cytomegalovirus, and with biological aging (35, 36). The observed association therefore suggests that cumulative BZ exposure may be related to disrupted T-cell homeostasis and is consistent with an immunosenescence-related alteration, although the CD4/CD8 ratio is not specific to immune aging. P53 is a central regulator of the DNA damage response, cell-cycle arrest, senescence and apoptosis (37, 38). The concurrent associations of higher CED with comet-assessed DNA damage and lower p53 expression suggest that cumulative BZ exposure may affect the regulation of DNA-damage-response pathways. This interpretation is consistent with previous reports of epigenetic dysregulation of tumor-suppressor genes in BZ-exposed populations (6, 38–40). However, the functional implications of lower p53 mRNA expression remain to be established. In contrast, the fully adjusted models showed no significant independent association between CED and CD28^−^ frequency, mtDNA copy number, or *γ*-H2AX expression. CD28 loss is an established marker of terminal T-cell differentiation and replicative senescence and is strongly influenced by chronological age (22, 41). Blood mtDNA copy number is likewise influenced by age, adiposity and circulating cell composition (42, 43), and previous BZ studies have reported inconsistent mtDNA findings, including increased copy number under low-grade exposure (44, 45). The absence of an association with *γ*-H2AX does not necessarily contradict the comet assay findings, because these assays reflect different aspects and temporal characteristics of the cellular response to DNA damage. Collectively, these null findings indicate that the investigated immune and cellular-stress markers did not respond uniformly to cumulative BZ exposure and caution against interpreting the observed alterations as evidence of generalized immunosenescence.

Although PLT and NEUT differed across the study groups, neither was significantly associated with CED after full multivariable adjustment. The association between CED and NEUT observed in the age- and sex-adjusted model was attenuated after further adjustment, whereas no statistically significant association between CED and PLT was observed in either model. Together with the null findings for WBC and RBC, these results do not indicate a consistent exposure-related alteration in routine peripheral blood counts in this cohort. Similar null or inconsistent associations between low-level BZ exposure and routine blood cell counts have been reported in some occupational studies (46, 47), whereas others have observed reductions in WBC, granulocyte, lymphocyte, and platelet counts at exposure levels below 1 ppm (12). The differences between these findings and our results may reflect variation in exposure intensity and duration, study-population characteristics, and control of potential confounders. The absence of consistent associations with routine blood counts does not exclude subclinical biological effects but indicates that these conventional measures did not closely reflect cumulative BZ exposure in the present study.

Importantly, occupational BZ exposure levels in this study were below the applicable regulatory limits, yet CED remained associated with comet-assessed DNA damage and selected immune-related alterations. In contrast, routine peripheral blood counts showed no consistent associations with CED after full adjustment. This pattern suggests that selected effect biomarkers may detect subclinical biological alterations that are not reflected by conventional hematological surveillance. However, the presence of biomarker associations below regulatory limits does not by itself demonstrate clinically significant harm or establish that the current limits are insufficiently protective. If validated in prospective studies, biomarkers of DNA damage and immune-related alterations could potentially complement routine blood counts and contribute to the earlier identification of BZ-related biological effects (48). Their specificity, reproducibility, predictive value, feasibility, and clinically meaningful thresholds must be established before they can be considered for incorporation into occupational health surveillance.

Despite these findings, several limitations should be considered. First, the cross-sectional design precludes causal inference and does not allow determination of whether the observed immune-related alterations represent accelerated aging relative to chronological age. A healthy-worker survivor effect may additionally have attenuated or distorted the exposure-response relationships (49). Second, although major lifestyle and demographic confounders were controlled, residual confounding cannot be excluded. In particular, genetic polymorphisms in BZ-metabolizing and DNA-repair genes (50), cytomegalovirus serostatus, which is an important determinant of CD4/CD8 ratio and T-cell differentiation (18, 51), and co-exposure to other workplace agents were not assessed. Exposure reconstruction was also subject to potential non-differential misclassification. Third, because the participating workforce was predominantly male, female participants were few, unevenly distributed across the study groups, and absent from the exposed group. Although sex was included in the multivariable models, this imbalance precluded reliable sex-specific analyses, may have limited control of sex-related confounding, and restricts the generalizability of the findings to female workers. Fourth, given the number of biomarkers examined, the findings should be regarded as exploratory, and the possibility of chance findings due to multiple comparisons cannot be excluded. In addition, MNF was determined by scoring 1,000 binucleated cells per participant. Because MN are relatively infrequent events, this scoring depth may have reduced the precision of MNF estimates and the statistical power to detect modest exposure-related differences. Finally, p53 expression was measured in whole blood without functional validation and may have been influenced by variation in circulating cell composition. MtDNA was assessed at the copy-number level without corresponding functional assessment. All biomarkers were measured in peripheral blood; although the bone-marrow niche is a primary site of BZ-induced hematopoietic injury, marrow sampling is generally not feasible in healthy working populations.

In conclusion, cumulative occupational BZ exposure below the applicable regulatory limits was associated with greater comet tail intensity and tail moment and with lower CD4/CD8 ratio and p53 expression after multivariable adjustment. The association between CED and MNF remained positive but did not reach conventional statistical significance in the fully adjusted model. These findings suggest that cumulative BZ exposure may be associated with selected genotoxic and immune-related alterations that are not reflected by routine peripheral blood counts, rather than with generalized hematological toxicity or a uniform immunosenescence phenotype. Prospective studies incorporating repeated exposure assessment, greater CBMN scoring depth, validated immune-aging biomarkers, and relevant host factors such as cytomegalovirus serostatus are warranted to clarify the long-term significance of these alterations and their potential value in occupational health surveillance.

## Data Availability

The original contributions presented in the study are included in the article/supplementary material, further inquiries can be directed to the corresponding author.
